# Aortic Dissection Case Report

**DOI:** 10.21980/J8964Z

**Published:** 2023-01-31

**Authors:** Chelsea Bunce, Christopher Bryczkowski, Mary Rometti

**Affiliations:** * Rutgers Robert Wood Johnson Medical School, Department of Emergency Medicine, New Brunswick, NJ

## Abstract

Although uncommon, acute aortic dissections are a life-threatening, cannot miss diagnosis for the emergency medicine clinician. Point of care ultrasound can play an integral role in the initial work up of the undifferentiated patient. While not initially utilized to make the diagnosis of aortic dissection, the ultrasound images obtained in this case describe key findings on ultrasound vital for an emergency clinician to recognize. It is essential for emergency medicine clinicians to differentiate an aortic dissection from other causes of chest pain and abdominal pain because the quick mobilization of resources plays a key role in the management and outcome of such patients.

**Topics:**

Aortic dissection, vascular, dissection flap, back pain, point of care ultrasound, POCUS.

**Figure f1-jetem-8-1-v5:** Video Link: https://youtu.be/iTA_ykjBxUo

**Figure f2-jetem-8-1-v5:** Video Link: https://youtu.be/s4IyVx4RxxI

## Brief introduction

[Fig f1-jetem-8-1-v5][Fig f2-jetem-8-1-v5]Acute aortic dissection is an exceedingly rare, but catastrophic disorder. Acute thoracic aortic dissection is associated with high morbidity and mortality rates.^1^ Complications of aortic dissection include aortic rupture, tamponade, and acute aortic valve insufficiency. The true incidence of aortic dissection is difficult to describe due to the high early mortality and morbidity rate and the challenge of diagnosing the disease.^1^–^3^ Ascending aortic dissections are almost twice as common as descending dissections. Data on the history, epidemiology, and population health issues related to dissection are limited.^4^ Physicians suspect the diagnosis in as few as 15% to 43% of verified cases of acute aortic dissection. If untreated, mortality approaches 50% in the first 48 hours.^2^,^5^ Point of care ultrasound (POCUS) is vital for helping to quickly diagnose aortic dissections from the bedside, especially if the patient is too unstable to transport to a computed tomography angiography (CTA) scan.

## Presenting concerns and clinical findings

A 75-year-old female initially presented to a community hospital with headache and a few days of left leg pain which radiated to her lower back. On the morning of her presentation, she reported right foot numbness and had multiple falls. At the outside hospital, imaging was performed which revealed an aortic dissection, triggering an emergent transfer to a tertiary academic hospital for definitive care.

During physical examination in the emergency department (ED) at the receiving facility, she was awake and alert. Her heart rate was 85 and blood pressure 107/66. The rest of her exam was significant for a large, midline, nontender, non-erythematous abdominal hernia. The abdomen was soft with moderate tenderness in the epigastric area. The patient was moving all extremities and distal pulses were intact. A review of her past medical history revealed a previous type A aortic dissection which was repaired, hypertension, and chronic obstructive pulmonary disease (COPD). She was on multiple medications, including aspirin and furosemide.

## Significant findings

In transverse view, point-of-care ultrasound (POCUS) showed an anechoic circular true lumen (blue highlight) and half-circular anechoic false lumen (green highlight), separated by a near hyperechoic dissection flap (orange highlight) that pulsated with blood flow. When viewed in sagittal orientation, the anechoic true lumen (blue highlight) appears longitudinal, separated from the false lumen (green highlight) by a dissection flap (orange highlight). Stills showing the measurements of these dissections are also provided.

## Patient course

In the ED at the accepting hospital, POCUS was performed revealing an abdominal aortic dissection, as described above. Fentanyl was given for pain control. The patient underwent operative vascular surgical repair of her aortic dissection with placement of a stent graft. She was admitted to the intensive care unit (ICU). On post-operative day (POD) 4, she developed hemodynamic instability and was intubated. Imaging revealed concern for possible aortic rupture, so she was taken back emergently to surgery. In the operating room (OR), vascular and general surgery attempted to repair the ruptured type b aortic dissection. Unfortunately, disease progression was extensive and there was concern for intra-abdominal compartment syndrome. The team discussed with the family and unfortunately the patient expired in the OR.

## Discussion

An aortic dissection occurs when the layers of the aortic wall separate. The wall of the aorta is made up of three layers - an inner layer (the intima), middle layer(media) and outer layer(adventitia).^3^,^6^ The dissection begins abruptly when a tear occurs in the inner layer of a weakened area of the aorta. When blood surges through the tear, the inner and middle layers separate or dissect. Most of the tears occur in the ascending aorta, usually in the right lateral wall where the greatest shear force on the aorta occurs. An acute aortic dissection can propagate anterograde and/or retrograde depending on the direction the dissection travels, causing branch obstruction that produces ischemia of affected territory (coronary, cerebral, spinal, or visceral).^6^ Proximal type A dissections can instigate acute tamponade, aortic regurgitation, or even aortic rupture.^6^

There are two main classifications used to characterize aortic dissections. First, the Sanford system, which is used more frequently, classifies dissections based on whether they involve the ascending or descending part of the aorta. Type A involves the ascending aorta, regardless of the site of the primary intimal tear. A type A dissection is defined as a dissection proximal to the brachiocephalic artery, while a type B dissection is defined as originating distal to the left subclavian artery and involving only the descending aorta.^3^,^5^,^6^ In contrast, the DeBakey classification system is based on the site of origin of the dissection. Type 1 originates in the ascending aorta involving at least the aortic arch. Type 2 originates in and only involves the ascending aorta. Type 3 begins in the descending aorta. Type 3a extends above the diaphragm and type 3b extends below the diaphragm.^3^,^6^

While CTA is considered one of the gold standards for diagnosing aortic dissections, point of care ultrasound offers a noninvasive way to begin diagnostic evaluation of a critical patient without leaving the bedside.^7^,^8^ In a study by Wang et al, emergency physicians used POCUS in patients with suspected aortic dissection compared to CTA, which revealed that POCUS had a sensitivity of 86.4% and a specificity of 100.0%.^9^,^10^ While ultrasound is helpful in diagnosing an aortic dissection, a negative bedside ultrasound does not completely exclude aortic pathology.^7^

When performing an ultrasound of the abdominal aorta, the curvilinear probe is typically used because it has a lower frequency and can more effectively image deeper structures to aid in viewing the aorta. In transverse view, the abdominal aorta should be imaged from the diaphragm to the point of aortic bifurcation into the common iliac arteries distally.^11^ Additional assessment of the aorta should be made in the sagittal plane too.^11^ To diagnose an aortic dissection on ultrasound, look for a dissection flap in either imaging plane, as annotated above on images from our case.^11^ For this diagnosis, there are limitations of using ultrasound, such as acquisition and interpretation of images being user-dependent, patient body habitus, and limited viewing field.^8^

Urgent cardiothoracic or vascular surgery consultation should be obtained once the diagnosis is made. Treatment of aortic dissections initially involves providing adequate analgesia and administering short acting IV beta blockers aiming for a heart rate of 60 beats per minute. Morphine is the preferred analgesic because it decreases sympathetic output. The reductions in heart and blood pressure from beta blockers reduces aortic wall tension and limits the extent of the dissection. The goal of the systolic blood pressure is 100 to 120 mmHg, and if beta blockers alone are unable to achieve this, then Nitroprusside or other antihypertensive agents can be added.^12^,^13^

Acute dissections involving the ascending aorta are surgical emergencies. Surgical therapy for type A involves the excision of the intimal tear, obliteration of entry into the false lumen proximally, and reconstruction of aorta with a synthetic vascular graft.^2^,^12^,^13^ Uncomplicated type B aortic dissections can be managed medically. Early endovascular intervention for uncomplicated type B aortic dissection is controversial. Intervention is generally reserved for those who progress to a complicated disease such as mal perfusion, uncontrollable pain, hypertension, expansion, rupture or impending rupture. Endovascular stent grafting has been evaluated for acute and subacute type B aortic dissection. Endovascular repair involves placing the stent graft over the intimal flap to seal the entry site of dissection resulting in thrombosis of the false lumen. Open surgical repair is rarely needed for acute type B dissections.^5^,^12^,^13^

This case highlights the importance of recognizing an acute aortic dissection quickly, so that time-sensitive interventions can be made. Rapid identification can improve patient outcomes and mobilize lifesaving resources. A limitation of this case is that it does not demonstrate bedside ultrasound’s utility in diagnosing aortic pathology before CT. While bedside ultrasound did not make the initial identification of an aortic dissection, the bedside ultrasound performed in this case depicts the value that POCUS can have in evaluating for this diagnosis among a broad differential. Unfortunately, in this case, the dissection had progressed, and the patient ultimately expired.

## Supplementary Information












