# Supplementary figures and images for: Aortic Dissection Case Report
Source: J Educ Teach Emerg Med. 2023 Jan 31;8(1):V5–V10. doi: 10.21980/J8964Z (PMC10332776; doi:10.21980/J8964Z)

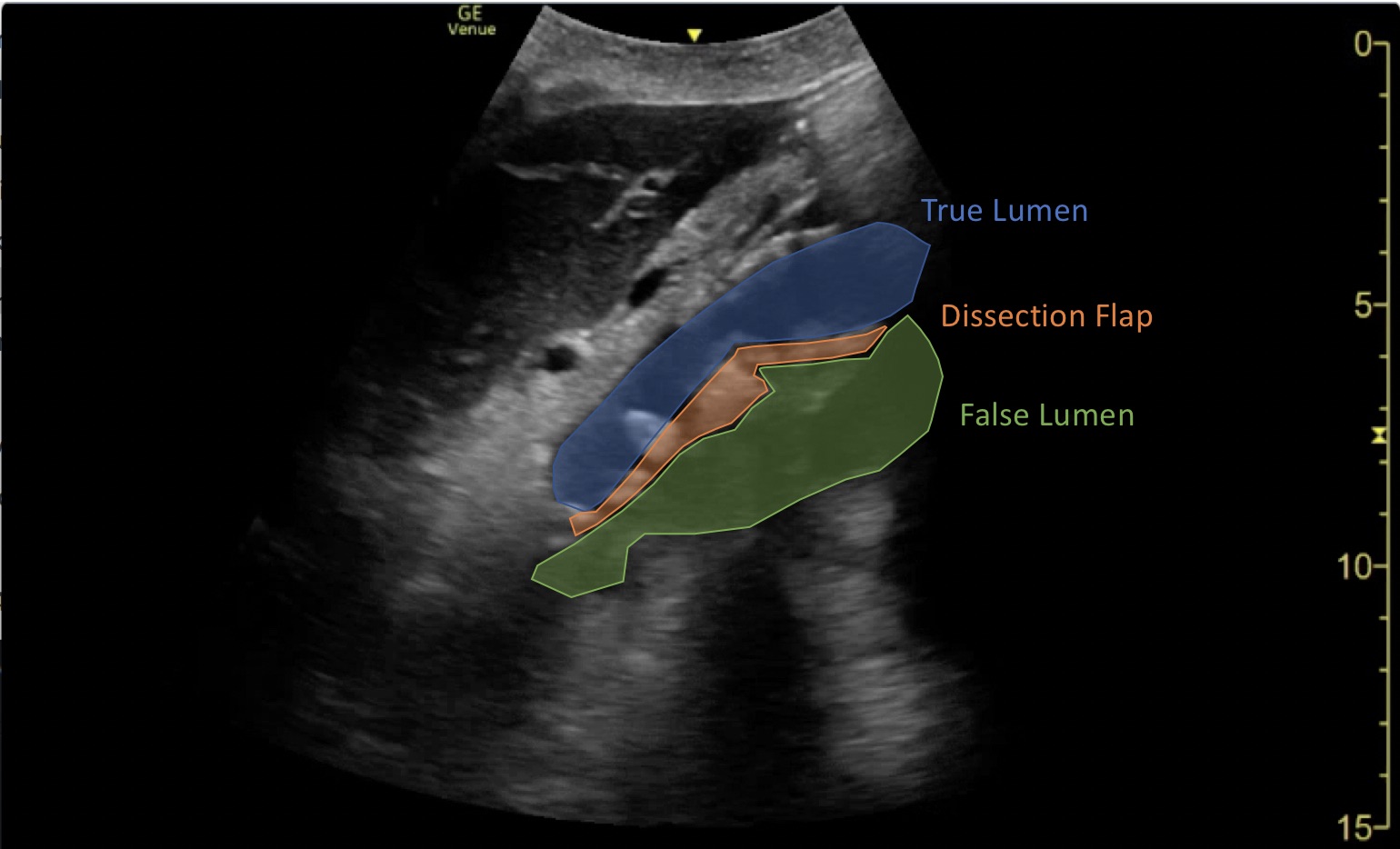

Supplement: Supplementary file 3 [file jetem-8-1-v5-supp1.jpg]

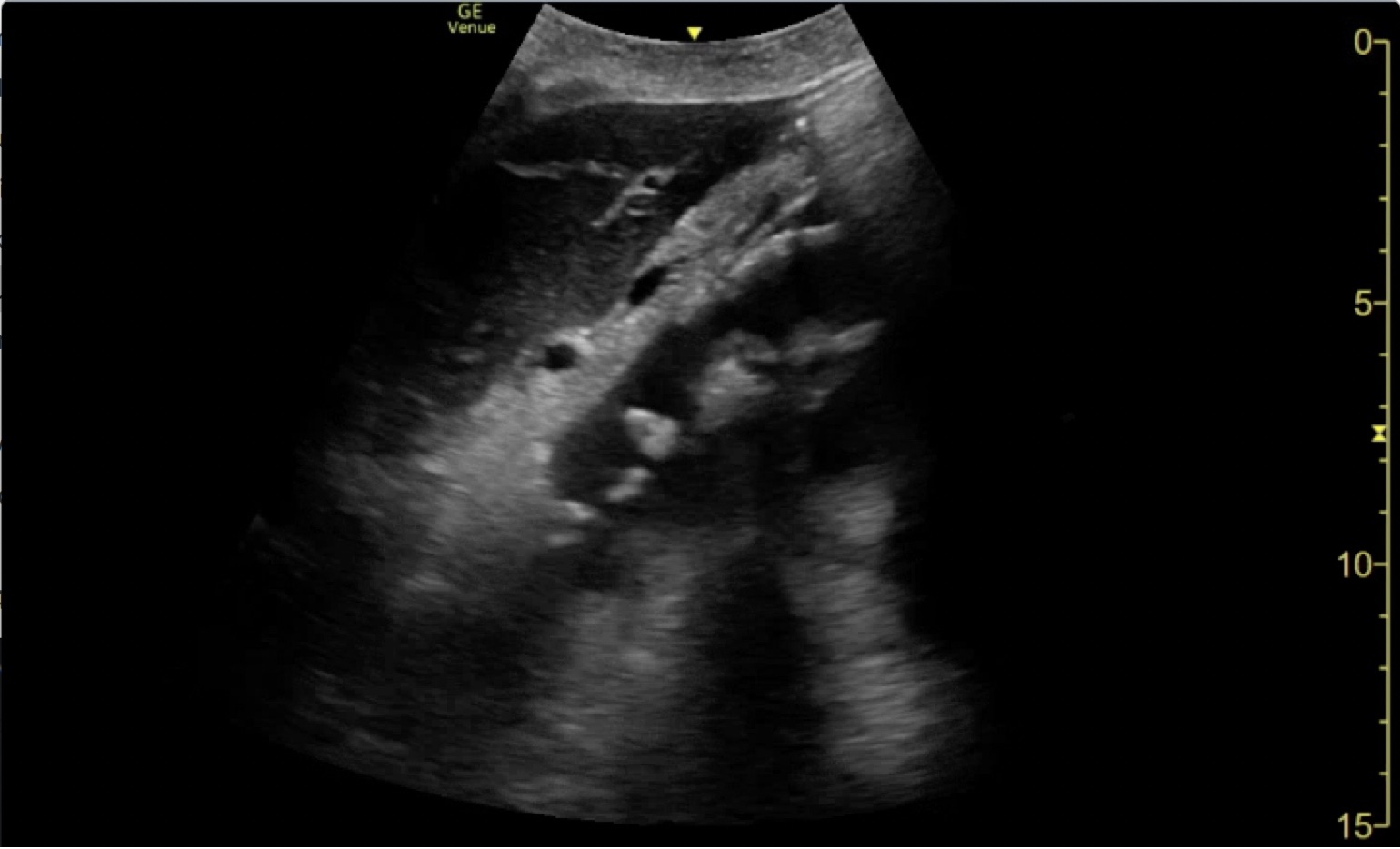

Supplement: Supplementary file 4 [file jetem-8-1-v5-supp2.jpg]

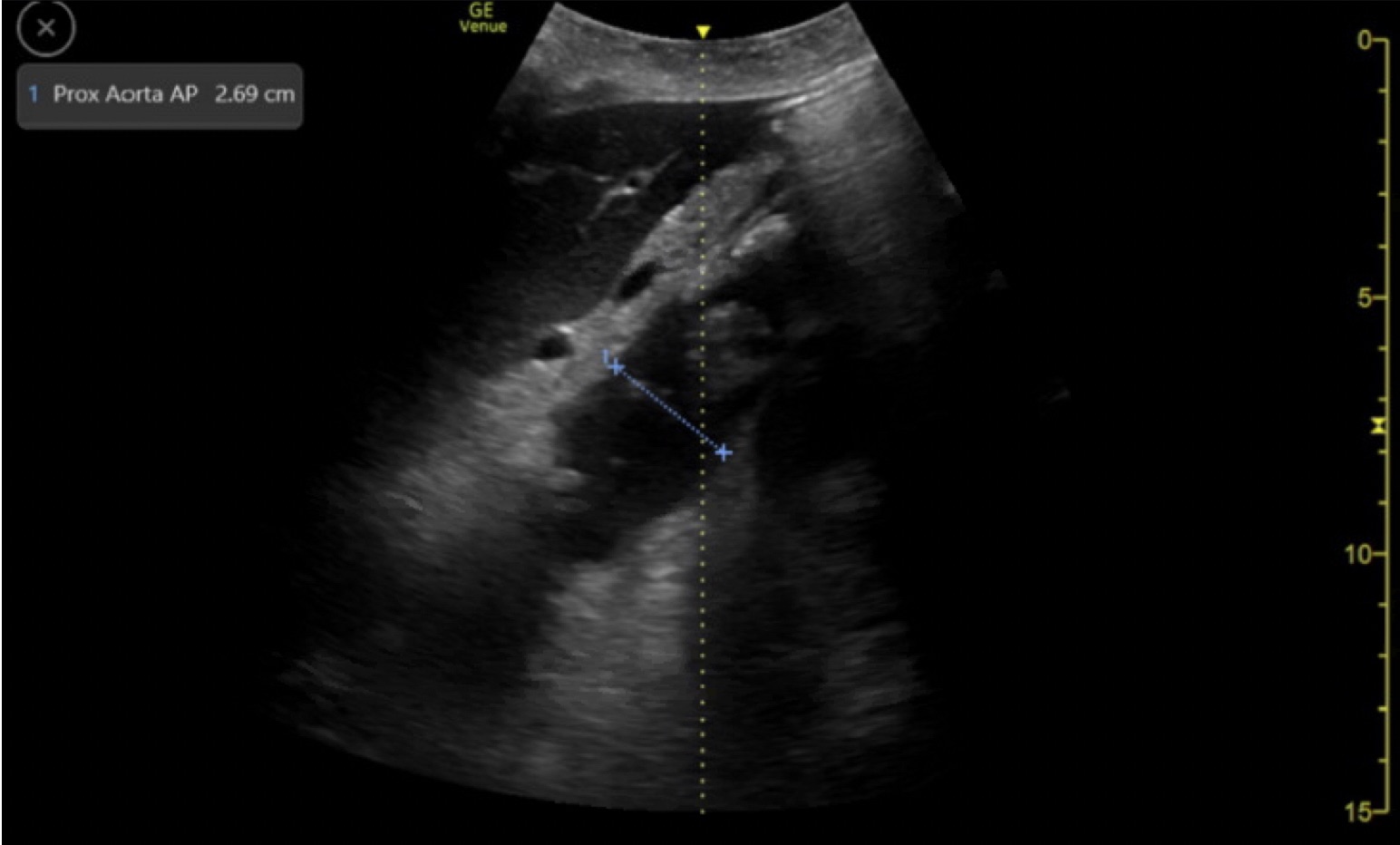

Supplement: Supplementary file 5 [file jetem-8-1-v5-supp3.jpg]

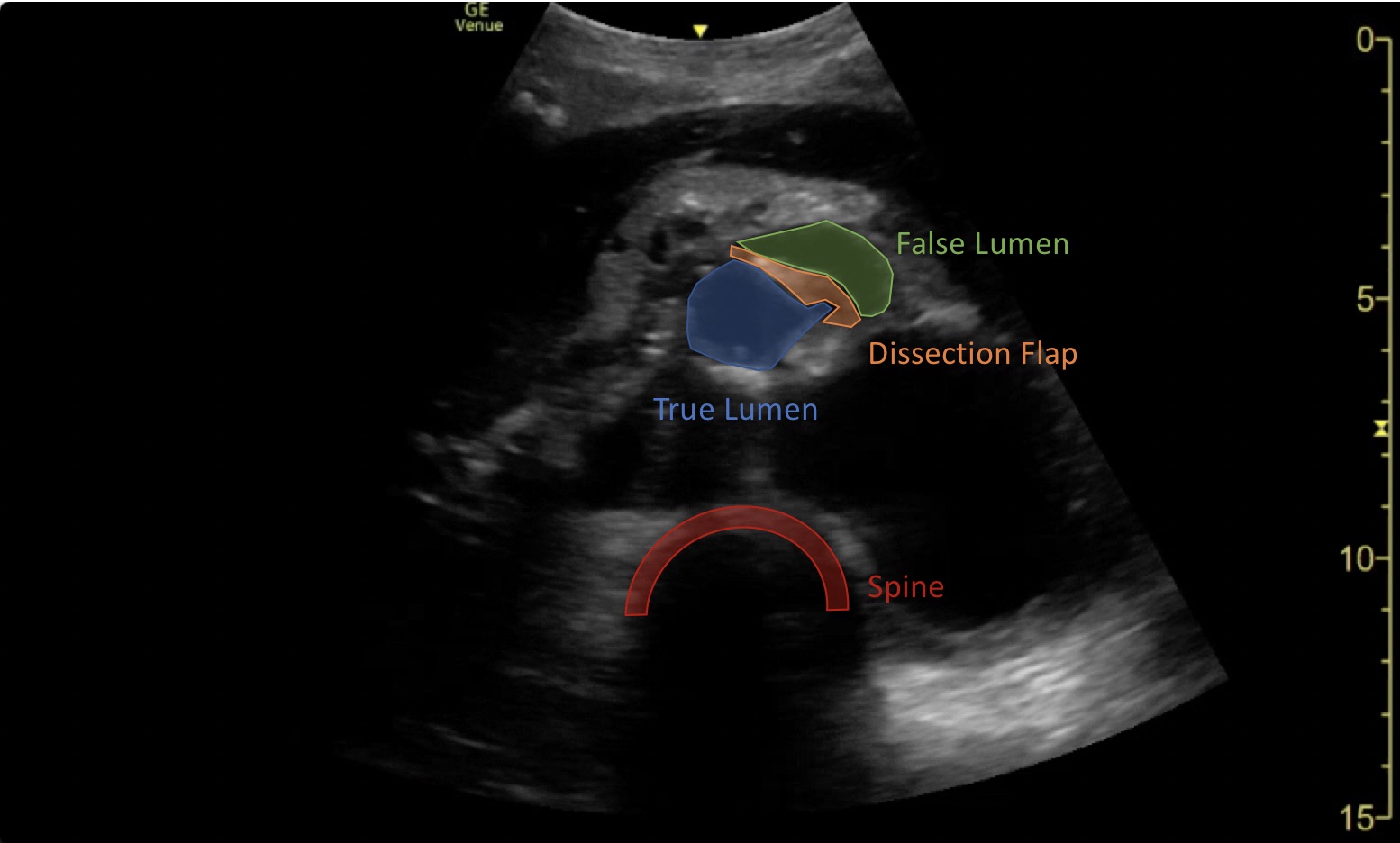

Supplement: Supplementary file 6 [file jetem-8-1-v5-supp5.jpg]

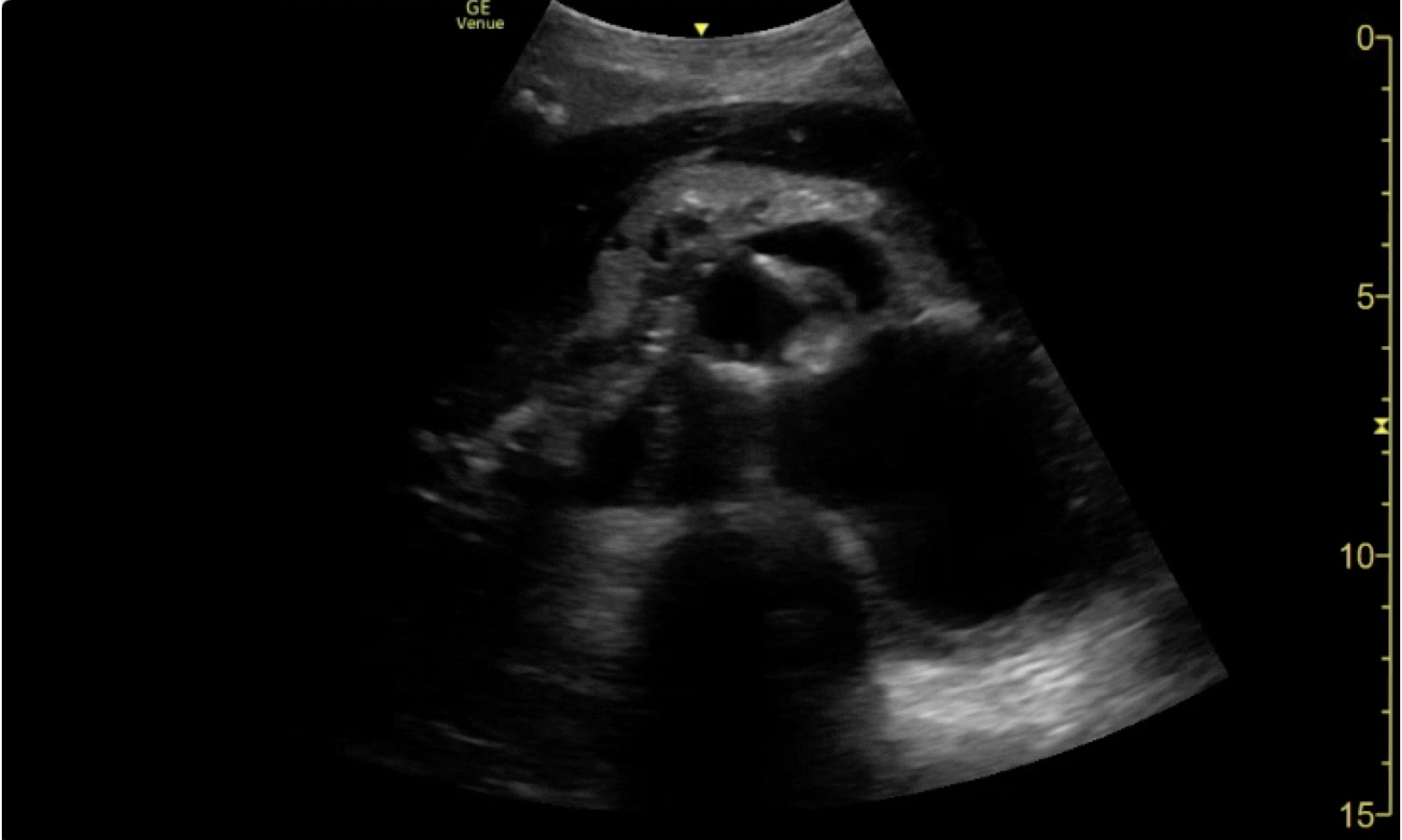

Supplement: Supplementary file 7 [file jetem-8-1-v5-supp6.jpg]

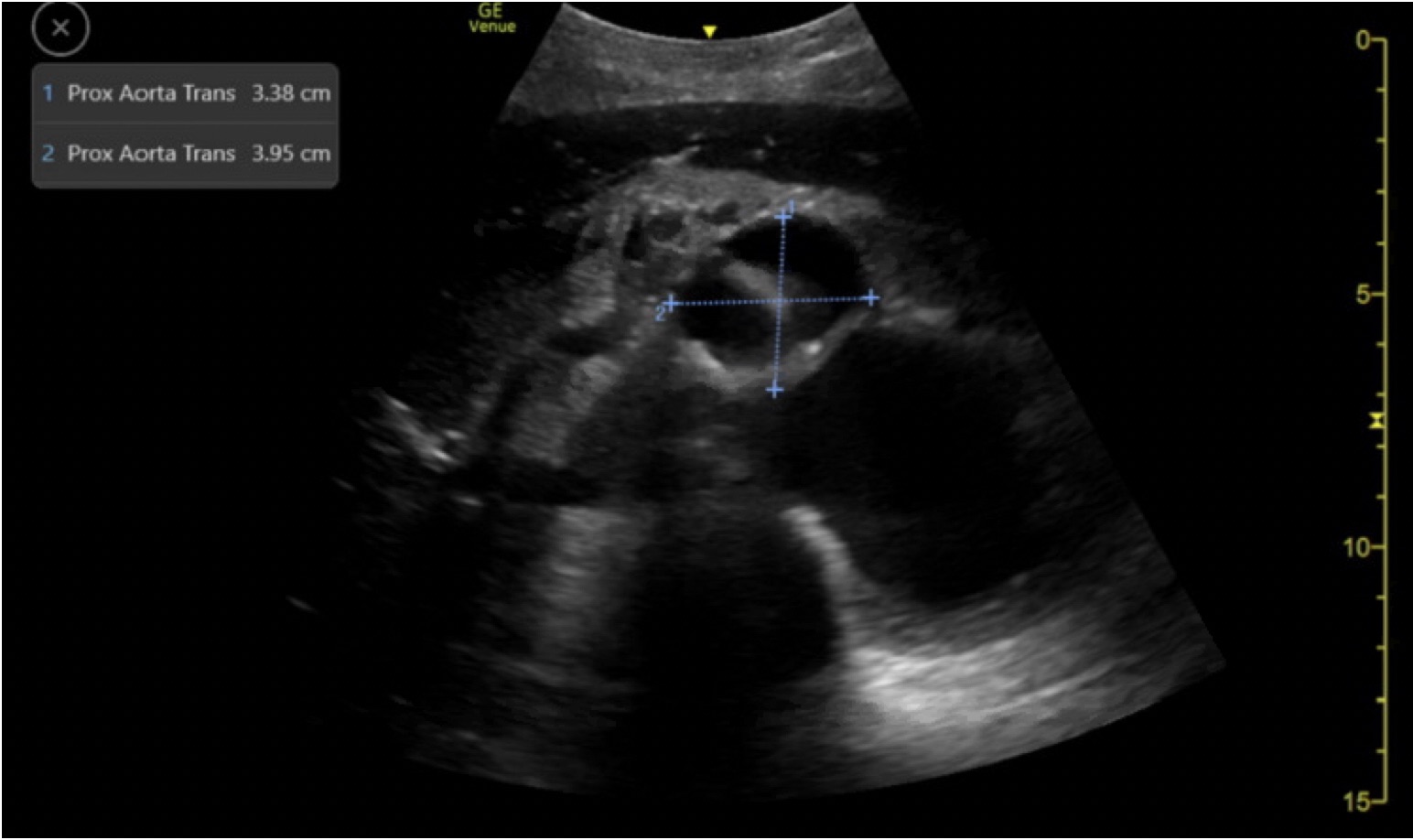

Supplement: Supplementary file 8 [file jetem-8-1-v5-supp7.jpg]
